# Does information improve service delivery? A randomized trial in education in India

**DOI:** 10.1371/journal.pone.0280803

**Published:** 2023-03-15

**Authors:** Priyanka Pandey

**Affiliations:** World Bank, Washington, DC, United States of America; Universidad de Murcia, SPAIN

## Abstract

From a cluster randomized control trial in 610 villages, the study evaluates the impact of a community-based information campaign on school outcomes in three Indian states. The campaign consisted of eleven to fourteen public meetings over two rounds in treatment villages to disseminate information to the community about its state-mandated roles and responsibilities in school management. No intervention took place in control villages. The paper reports on the final follow up survey two and half years after the campaign. Providing information improved teacher effort and learning outcomes in schools. Bigger gains were seen in the two states, Madhya Pradesh (MP) and Uttar Pradesh (UP), where baseline level of outcomes was lower than in the third state, Karnataka. The impact on teacher effort, primarily for civil-service teachers with permanent jobs and therefore lower accountability, was between 16%-43% in MP and UP. The shares of children able to do basic mathematics competencies improved. Due to low baseline learning levels, the magnitudes of the percentage increases were much larger compared to the absolute increases in shares. Fewer improvements occurred in language. This can be because of low teaching effectiveness as well as more time needed for larger and wider impacts on learning. School councils became more active after the campaign. Focus group discussions indicated discussions within communities and communities actively bringing up issues with teachers and school councils. Impacts were generally larger or broader than those at midline survey 2–4 months after one round of intervention. Overall providing information holds promise in improving public services via worker accountability.

## Introduction

Failure to learn is common in public schools in developing countries plagued by low quality education despite the progress made in improving access to school [1]. For example, India ranked second from bottom in an international test of 15-year olds in 73 countries [2]. Almost 85% of grade 2 children could not read a single word of a short text and 86% could not perform two-digit subtraction in rural India [1]. Only about half of primary school completers in India could read a three sentence passage [3]. For an individual, poor education means significantly diminished life-chances of attaining social, material and physical well-being [1].

The problem of poor quality outcomes is a more general one, not limited to education or to India alone, and is linked to poor delivery of services [1, 4, 5]. Targeting resources efficiently to communities and ensuring public workers perform continues to be a challenge in many countries [4, 6, 7]. Reasons for deficient service delivery include inadequate resources and weak accountability mechanisms [5, 8, 9]. Despite their high absence rates in several countries, public workers such as those in health and education rarely face censure for failure to show up and do their jobs [10–12]. For example, 23% to 57% of teachers were absent from school or from classrooms while at school across several countries [1]. Physical resources alone cannot improve outcomes when public workers don’t deliver as expected.

Intended to increase service providers’ accountability to the local community, India like a number of developing countries has decentralized control over public services to local communities [8, 13]. However evidence suggests communities are uninformed of what services they are entitled to and what state-mandated controls they have over these services [6]. Local governments and their oversight committees meet infrequently and council members are often unaware of service delivery issues [14–21]. Parent members might not know of their membership and even though meetings are shown as held, members are unaware of them. Widespread poverty, with about 1 in 5 Indians living on less than $1.90 a day, illiteracy, with only 74% of Indian population literate, and social divisions like caste make individual or collective action to improve service delivery difficult in such settings [22, 23].

One way to enhance accountability may be to provide information that empowers stakeholders to demand quality services [24–26]. A newspaper campaign in Uganda that publicized diversion of primary school funds led to a reduction in embezzlement from 80% to 20% which had a positive effect on learning outcomes [27, 28]. However, evidence from informational interventions that involve random assignment to intervention and rigorous evaluation of outcomes is limited and mixed [29–32]. Results from the studies are not strictly comparable due to differences in the structure of information dissemination, outcome measurement, and follow-up period. A lesson thus far is that to inform policy on the efficacy of information-based interventions, research needs to be based on replicable means of information dissemination, objective outcome measurement and longer follow-up time period as behavioral change that improves outcomes can take time.

We conducted a community-based randomized controlled trial to determine the impact of information delivery on education outcomes. The campaign gave information to parents on their oversight roles in schools and education services they are entitled to. A key feature of the campaign was community-wide big public meetings that parents, teachers and school committee were invited to attend. Over a period of two and a half years and two rounds of intervention, 11–14 public meetings took place in each treatment village. Our hypothesis was if communities are empowered with this information, outcomes may improve.

The study was conducted in three Indian states—Karnataka, Madhya Pradesh (MP) and Uttar Pradesh (UP). An amendment to the Indian Constitution in 1992, the 73^rd^ amendment, mandated Indian states to devolve control over public services and their finances to local communities, leaving up to the states how much control to devolve [33]. Further policy initiatives such as the Right to Education Act passed in 2009 made school councils mandatory in public schools. The three states differ in the extent of oversight roles devolved to the community. MP and UP are two large north Indian states that lag in economic and social outcomes, while Karnataka in southern India is economically and socially more ahead in most development indicators. MP and UP are among the eight poorest states, out of 35 Indian states, that had more poor people (421 million) than the 26 poorest African countries combined, based on a 2010 multidimensional measure of poverty covering a range of deprivation including health and education [34]. In more recent data, MP and UP have around 40–41% of population that is multidimensional poor and are among the 5 poorest states while Karnataka has about 17% of population multidimensional poor [35]. In rural areas where 70% of the Indian population live, illiteracy and poverty are greater (S1 Table) [23, 34, 35]. Nearly one-third of teachers were absent on any given day, and more than 70% of public school children fail to acquire minimal reading, writing or mathematics skills by grade 4 in MP and UP, while Karnataka, had higher levels of both teacher and student outcomes [36, 37].

The size of the impact of such an intervention will depend on several factors. First, changing attitudes and behavior require time, so time elapsed between the intervention and follow up survey is important. The extent of oversight communities have over schools and extent of public action in response to the campaign will also matter. The impact of the campaign at midline 2–4 months after one round of the intervention showed an increase in teacher effort and modest gains in learning [32]. But the impact differed across states possibly due to different starting points at baseline and variation in the extent of devolution. This paper reports findings at end line after two rounds of intervention and two and half years after initial intervention.

## Methods

### Setting

The study is a cluster randomized control trial (RCT) of 610 *gram panchayats* (GPs) in three states, Karnataka, MP and UP- randomly allocated to receive the treatment which is an information campaign or serve as control. A GP is a cluster of approximately 1–3 adjacent villages and is the smallest unit of local government that consists of an elected head and council members. The terms GP and village used interchangeably in the paper, in all cases mean the former.

The trial was conducted from 2006 to 2009. Four districts were chosen purposefully in each state, matched across states by literacy rates so that they are comparable on at least one indicator of development. Within a district, 50 GPs were selected from two randomly chosen blocks. A block is the administrative unit between a district and a GP. We used a random number generator to randomly select blocks and then GPs within the blocks. Half of the GPs within each block were then randomly assigned to intervention arm and the remaining half to control arm. Treatment and control GPs were evenly spread across the two blocks to reduce any potential contamination. This gives a total of 100 control GPs and 100 treatment GPs per state in MP and UP.

In Karnataka the design was identical except an additional set of treatment villages was added that received a slightly different treatment called information and advocacy campaign. The number of GPs in each of the three cells in Karnataka, control, treatment 1 (information campaign), treatment 2 (information campaign plus advocacy), totaled 70.

Sample sizes were determined using cluster randomized sample size calculations based on a 5 percent significance level and 80 percent power.

### Timeline and steps of the study

#### Timeline

In MP and UP, baseline surveys were administered between February-April 2006. The first round of intervention was carried out from September 2006 to January 2007, and the first follow-up surveys administered between February and April 2007. A year after the baseline survey, focus group discussions were held in select intervention GPs. A second round of intervention was from September 2008 to January 2009, with second follow-up surveys between February and April 2009. In Karnataka, baseline surveys were administered between July and August 2006. The first round of intervention was from February to May 2007, and the first follow-up surveys between July and August 2007. The second round of intervention was from February until May 2009, and second follow-up surveys administered between July and August 2009. Karnataka had a shifted timeline due to difference in school year timing.

#### Baseline survey

In each GP, one school was randomly selected from all public schools that had grades 1 to 5. All grades 1–5 teachers were in the sample. In MP and UP, 15 students were randomly selected from each of the grades 2, 3 and 4, totaling 45 per school. In Karnataka, 30 students were selected per school, 15 each from grades 4 and 5. School enrollment registers were used for random selection of students. In the case that a given grade had less than the required number of students, all students were selected.

The surveys were conducted via in-person interviews by a team of trained research assistants with prior experience in administering rural household surveys in the region. Surveys were conducted in the local language, Hindi in MP and UP, and Kannada in Karnataka, and instruments pilot tested prior to use.

#### Outcomes measured at baseline

Teacher attendance and activity: Four unannounced visits were made, one every 2–3 weeks, to record attendance and activity. Activity is a measure of whether a teacher was actively engaged in teaching when the team arrived. Attendance takes the value 1 if a teacher was present in school, 0 otherwise. Activity is 1 if the teacher was teaching, writing on the board, supervising written work, teaching by rote or another method, 0 if the teacher was absent, chatting, sitting idle or standing outside the classroom, keeping order in the classroom but not teaching, engaged in non-teaching work. Teacher attendance and activity variables are constructed as averages over the four visits for a teacher and interpreted as fraction of visits the teacher was present (or engaged in teaching). Both variables take values between 0 and 1.

*Learning*. Students were assessed on a competency based language and mathematics test that lasted approximately 20 minutes. The language test had reading and writing competencies. The mathematics test included addition, subtraction, multiplication and division. The competencies tested fell within or below those listed for the grade in the minimum level of learning (MLL) framework outlined by the Indian government. Learning outcomes are the shares of children who attain specific competencies: Percent of children who can read sentences and words, percent of children who can write sentences and words, percent of children who can do addition, subtraction and less (addition), multiplication and less (addition and subtraction), division and less (addition, subtraction and multiplication).

School council members were interviewed on their participation in school oversight activities and awareness of roles and responsibilities.

#### Intervention

The intervention was an information campaign. The tools used in the campaign consisted of a short film of 6 minutes, poster, wall-painting, take-home calendar and learning assessment booklet. The tools were same across states, except the information contained was state-specific. There are state-specific differences in the nature and extent of devolution, that is, in the structures created for the community to exercise local governance of schools and the amount of oversight devolved (described in S1 Appendix). The information was acquired from the states’ education offices, and tools verified and approved for their content by each state government prior to the campaign.

The film, poster, wall-painting and calendar focused on the following information: details of roles and responsibilities of school councils; rules for selection of council members; rules for council meetings including number of mandatory meetings, minimum attendance required in meetings and record keeping of minutes; organization and funding of school accounts; right to information with respect to schools including right to obtain copies of any school record; where to complain about any problems; benefits that students in primary grades are entitled to such as cash stipend, textbooks, mid-day meal, school uniforms. The film, poster and wall-painting contained key information highlights (S2 Appendix has an example of the information highlights) while the calendar contained all of the information in detail. The learning assessment booklet outlined the minimum levels of language and math skills that children were expected to acquire by grade, based on the MLL framework recognized by the government. The booklet was an easy-to-use tool for parents to assess whether their child knew the minimum expected for his or her grade.

In addition to the information campaign treatment in the three states, there was a second treatment only in Karnataka. This was a slightly different treatment with an additional 2 minute capsule at the end of the film which showed average wages for different levels of schooling to increase awareness of the economic benefits of schooling and advocated the audience to become involved in monitoring school outcomes. All other tools were the same as in the first treatment.

Campaign teams were blind to baseline and follow up surveys. The first round consisted of three visits to a GP, each visit separated by a period of two-three weeks. A key feature of the campaign was community-wide big public meetings that parents, teachers and school committee were invited to attend. On each visit, the team organized 2–3 such meetings across different neighborhoods of the GP. The target audience comprised of parents, school council members, and teachers. Separate meetings were held in low- and high- caste neighborhoods. The invitations were broadly publicized 2–3 days in advance via an audio track, the local village announcer and enlisting the primary school teachers’ help.

A meeting lasted about 40 minutes during which the film was screened twice followed by opportunity to ask questions, and discussion among the audience. To ensure uniformity, research assistants followed a script, read out the introduction and were only allowed to reply to questions to which the answers were already written on the calendars. Any other questions or issues raised were not answered. The team was instructed to not participate in any discussion that took place among the community members following the film presentation. The audience was told that the information was collected from the government.

After a meeting, calendars and learning assessment booklets were distributed door-to-door. Posters and wall-paintings were displayed in the school and other prominent locations.

The second round was similar to the first—each GP visited thrice and visits separated by a period of three-four weeks. A focus group discussion in a small sample of treatment villages (see below) between the two intervention rounds informed the following changes based on suggestions from participants in the way meetings were organized without changing anything substantial. One change was that meetings were now mainly held at the school. If members from some caste groups or women were not willing to come to the common meeting due to village norms, then the team would hold another meeting in their neighborhood. A second change was that the film was excluded the last 2 times meetings were held. With the same film shown repeatedly, it was difficult to attract audience for the meetings. A third change was that in order to inform the illiterate audience, key information in the calendar was read aloud exactly as written to preserve uniformity. The campaign ended with 11 to 14 repeat meetings in every village cluster or GP over the two and half year period.

#### Follow-up survey

Baseline survey participants were re-interviewed after 12 months (midline or first follow-up survey) and 36 months (second or final follow-up survey) by research assistants who had no knowledge of the intervention. To maintain this blinding, intervention group teachers, students and council members were not asked if they had attended any informational meetings.

*Midline*. In MP and UP, sample students in grades 2–4 at baseline were followed and were now in grades 3, 4, and 5 respectively. In Karnataka, baseline sample students, who were now in grades 5 and 6, were followed. Each cohort by grade was administered the same test form as in baseline. For example, grade 3 students were given the same test form administered to them in grade 2 at baseline.

*Final follow-up*. In MP and UP, sample students in grades 3 and 4 at baseline (or in grades 4 and 5 at midline) could not be followed since they were out of the primary school cycle at this time. A fresh sample of students in grades 3, 4 was randomly chosen, while those currently in grade 5 were followed from midline and baseline (at which time they were in grades 3 and 2 respectively). In case a current grade 5 student followed from midline no longer was in school, the research team traced him or her in the village to administer the test. In Karnataka, sample students at baseline were out of the sample schools by this time. So a fresh sample of students in grades 4, 5 was chosen.

#### Focus group discussions (FGDs) and qualitative interviews

One year after the first round of intervention, we conducted FGDs in 10 randomly selected intervention GPs each in MP and UP. Two focus group meetings were held in every GP, one among residents from disadvantaged social classes or low caste (S3 Appendix has definition of low caste groups) and the other among non-low caste or high caste residents. Participants were asked if they remembered the campaign, post-campaign whether they had discussed the information with anyone in the village, whether they had raised school-related issues with teachers or school committees, why bigger changes were not seen and what could be done to improve the campaign.

Alongside FGDs, individual school committee members, 32 in UP and 50 in MP, were interviewed in the same GPs on questions posed to the focus groups, and a few additional questions on participation in school oversight. Note that the focus group was not conducted in Karnataka due to reasons of limited resources.

### Analysis

This paper reports impacts based on the final follow-up survey. The unit of analysis for teacher attendance and activity outcomes is the individual teacher. The unit of analysis for learning outcomes is the grade. For outcomes from interviews of school committee members, the unit of analysis is the school as explained below.

To measure the impact of the campaign, for each outcome we conduct a linear difference in differences regression analysis comparing the change in intervention to the change in control group from baseline to final follow-up after adjustment of standard errors for clustering. Comparable changes between baseline and midline are reported in previous work [32]. We used the regress and cluster commands from Stata 13.1 statistical software (StataCorp, College Station, Texas) for the analyses. Significance level is set at 5 percent. Weak significance level is set at 10 percent.

Focus groups and qualitative interviews are analyzed by the percentage of respondents to each question. Responses to open-ended questions are presented by main themes mentioned.

### Ethics statement

The World Bank does not have an ethics committee to review research proposals, nor does it use an internal review board [38]. However, the Bank reviews all research by its staff as part of the funding process. Approval for the study was obtained from the Ministry of Human Resource Development, Government of India. Before conducting the surveys, we first approached the locally elected village head and the school principal with the state permission and obtained permission from them. The Bank does not require signed agreement statements by subjects [38], and it would not be practical in our case since some are illiterate. Oral consent was obtained from all participants and in case of students, from at least one parent before administering the survey. It was explained to all participants that the study was about social behavior and education in villages. All participants were told that they were free to leave at any time. Participants were told that no personal information about them would be shared with anyone except the research team. Data are analyzed anonymously.

### Inclusivity in global research

Additional information regarding the ethical, cultural, and scientific considerations specific to inclusivity in global research is included in the Supporting Information (S1 Checklist).

## Results

### Baseline characteristics

Treatment and control villages are similar in socio-economic, demographic and other characteristics of the sample (S2 Table). In MP and UP, although baseline survey indicates no significant differences between treatment and control groups with respect to outcomes (Table 1), it highlights low teacher effort and poor learning. School council members were relatively inactive in school affairs (Table 1) and not well informed of their roles (S3 Table). When asked to list the council’s roles and responsibilities, 52% of parent members in UP and 58% in MP could not list a single one. There is substantial variation in outcomes across states (S2 Table and Table 1). Karnataka had much higher levels of teacher effort and student learning outcomes than the other two states, although some of the learning outcomes are higher in control relative to treatment groups in the state (Table 1). Average teacher attendance was 87% and average rate of teaching activity was 68%. Nearly 44–47% of students could read simple sentences and words (compared to 12–16% in the other two states). Although participation of school council members’ is similar to that in the other two states (Table 1), members’ have higher level of awareness (S3 Table).

**Table 1:** Baseline outcomes by randomized treatment status in MP, UP and Karnataka.

State→	UP			MP			Karnataka		
	Control	Treatment		Control	Treatment		Control	Treatment	
**Teacher effort**	n = 350	n = 338	P^a^	n = 239	n = 225	P^a^	n = 266	n = 599	P^a^
Teacher attendance	.68	.63	0.10	.69	.64	0.39	.86	.87	0.56
Teacher activity	.27	.26	0.68	.32	.29	0.12	.69	.67	0.45
**Learning**	n = 197	n = 200	P^a^	n = 196	n = 200	P^a^	n = 137	n = 272	P^a^
Language Read sentences and words	.12	.14	0.48	0.16	0.14	0.42	0.47	0.44	0.57
Write sentences and words	.06	.05	0.43	0.08	0.07	0.47	0.34	0.31	0.18
Math Divide, and less	.05	.04	0.57	0.05	0.05	0.93	0.32	0.26	0.09
	n = 297	n = 300	P^a^	n = 294	n = 299	P^a^	n = 137	n = 272	P^a^
Math Multiply, and less	.06	.06	0.80	0.08	0.10	0.20	0.39	0.31	0.06
Subtraction, and less	.10	.11	0.54	0.16	0.16	0.73	0.58	0.50	0.06
Addition	.18	.19	0.41	0.33	0.32	0.38	0.77	0.73	0.20
**School council**	n = 288	n = 273	P^a^	n = 266	n = 265	P^a^	n = 152	n = 348	P^a^
Number of meetings in past 1 year	1.24	1.15	0.31	2.34	2.39	0.40	1.62	1.80	0.16
Share of members who attended	0.54	0.51	0.19	.63	.64	0.80	.60	.68	0.054
Number of school visits in past 1 year	1.38	1.35	0.76	1.94	1.91	0.75	1.56	1.75	0.19
Share of members who participated in school visits	0.45	0.42	0.27	.64	.61	0.23	.55	.64	0.03
Total number who attended school visits	1.88	1.66	0.16	2.68	2.64	0.89	3.82	4.63	0.064

Mean values represent shares except indicated otherwise. Teacher variables are at individual teacher level, learning variables represent share of children aggregated at grade level, and school council variables represent member response aggregated at school level i.e., village cluster level by member type (chair, secretary, parent member).

^a^ P value for difference between treatment and control groups is based on clustered standard errors.

### Impact of the information campaign

The analysis is based on a “difference-in-differences” linear regression where the change in outcome from baseline to follow-up is the dependent variable. For individual *i* in GP *j* in block *k* in district *h*, the regression equation is as follows:

$$\Delta {ϒ}_{ijkh}=a+b\;X_{jkh}+b_{h}X_{jkh}*d_{h}+d_{h}+\varepsilon_{ijkh}$$

*ΔΥ*_*ijkh*_ is the change in outcome and *Χ*_*jkh*_ is a treatment dummy variable taking value 1 if village *j* in block *k* in district *h* belongs to the treatment group and 0 if it belongs to the control group. *d*_*h*_ are district dummy variables, *ε*_*ijkh*_ is a random error term and *a* is a constant term in the regression equation. The treatment effect is allowed to vary across districts. The estimate of the average treatment effect is the sum of the coefficient estimates of *b* and ∑_*h*_ *b*_*h*_ * *d*_*h*_ where district dummy variables *d*_*h*_ take the mean value in the sample.

Because the baseline outcomes are at different levels in Karnataka compared to MP and UP, the expected impacts are likely to be different. So results from MP and UP are discussed in one subsection and from Karnataka in a following subsection.

### Findings on impact in UP and MP

#### Teacher effort

In UP, the intervention is associated with 12% (8 percentage points; of 68 percent at baseline) increase in teacher attendance between baseline and follow-up (Table 2). At midline the impact was similar in that it was on teacher attendance and similar in magnitude [32]. In MP, the intervention is associated with increases of 9% (6 percent points; of 69 percent at baseline) in teacher attendance and 25% (8 percent points; of 32 percent at baseline) in teacher activity (Table 2). At midline the impact was only on teacher activity in MP although similar in magnitude [32]. When we separate the sample by tenure type—into civil-service and contract teachers—the impact is primarily on civil-service teachers in both states. Civil-service teachers have permanent jobs which means greater job security and lower incentive to work compared to contract teachers. In UP, their attendance increased by 23% (14 percent points; of 61 percent at baseline). In MP, civil-service teachers’ attendance and activity increased by 16% (11 percent points; of 67 percent at baseline) and 43% (13 percent points; of 30 percent at baseline) respectively. In comparison, at midline the impacts on attendance in UP and activity in MP were similar regardless of contract type [32].

**Table 2:** Difference-in-differences linear regression results in MP and UP where change in outcome (teacher effort) from baseline to follow-up is dependent variable.

State→	UP			MP		
Teacher effort	Treatment-Control (95% CI)	P	n	Treatment-Control (95% CI)	P	n
Attendance all teachers	0.08* (-.002 to 0.15)	0.05	474	0.06** (.01 to .10)	0.02	301
Activity all teachers	0.03 (-.02 to .08)	0.21	474	0.08** (.01 to .15)	0.03	301
Attendance civil service	0.14*** (.06 to .21)	0.00	184	0.11*** (.05 to .16)	0.00	251
Activity civil service	0.01 (-.03 to .04)	0.64	184	0.13*** (.07 to .18)	0.00	251
Attendance contract	0.03 (-.05 to .10)	0.44	290	-0.14 (-.40 to .11)	0.27	46
Activity contract	0.04 (-.04 to.13)	0.29	290	-0.14 (-.37 to .07)	0.18	46

Value represents coefficient on treatment variable. 95% confidence interval in parentheses.

***P < 0.01,

**P < 0.05,

*P < 0.10 based on clustered standard errors.

Disaggregating the analysis by characteristics of civil-service or permanent teachers, the impact is significant and greater in magnitude for high caste, male, and more educated teachers (S4 Table).

These findings can be due to increased monitoring by the communities as we find below. The extra scrutiny is likely to have affected those civil service teachers more whose motivation and effort levels were lower at baseline. Contract teachers, on the other hand, had higher effort rates, 16–22% higher attendance and 37–64% higher activity rates compared to civil service teachers at baseline [37]. They also had much lower salary levels, between one-fourth and one-fifth of civil service teachers’ salary [39]. This could explain why the initial impact on contract teachers observed at midline faded. It may not have been worthwhile for them to increase effort in the long(er) run, as not doing more would not invite social censure because in the community’s perception they may have been reasonably ‘maxed’ out.

#### Learning

As explained in Methods section, the unit of analysis for learning outcomes is the grade because at follow-up the sample of grades 3 and 4 students is a fresh sample while grade 5 sample had participated in baseline. Therefore in learning outcome regressions we include an interaction term of a dummy variable representing grade 5 interacted with the treatment dummy variable. Results are reported accordingly, and coefficients on treatment variable are presented for grade 5 and grades 3/4 separately in the table below.

We find a consistent impact mainly in the share of children acquiring mathematics competencies (Table 3). In UP, increases are observed in the percent of children who can do addition (3 percentage points; 20% of baseline average of 15 percent), subtraction and less (2 percentage points; 25% of baseline average of 8 percent), multiplication and less (2 percentage points; 40% of baseline of 5 percent), and division and less (3 percentage points; 73% of baseline of 4 percent) in grades 3 and 4. In grade 5, increases are in the percent of children able to do addition (4 percentage points; 15% of baseline of 26 percent), and subtraction and less (3 percentage points; 20% of baseline of 15 percent).

**Table 3:** Difference-in-differences linear regression results in MP and UP where change in learning outcome from baseline to follow-up is dependent variable.

State→	UP					MP				
Learning outcome	Grade 5^a^		Grade 3/4^b^			Grade 5^a^		Grade 3/4^b^		
	Treatment-control (95%CI)	P	Treatment-control (95%CI)	P	n	Treatment-control (95%CI)	P	Treatment-control (95%CI)	P	n
Read sentences and words	0.01 (-.02 to .05)	0.27	0.05* (-.001 to .11)	0.053	394	-0.01 (-.04 to .03)	0.70	0.02 (-.06 to .12)	0.51	396
Write sentences and words	0.00 (-.04 to .04)	0.99	0.03** (.01 to .05)	0.01	394	0.03 (-.03 to .08)	0.30	0.00 (-.04 to .05)	0.83	399
Divide, and less	0.02 (-.02 to .05)	0.22	0.03* (.0003 to .05)	0.048	394	0.02** (.0002 to .03)	0.05	0.00 (-.06 to .06)	0.93	396
Multiply, and less	0.01 (-.009 to .05)	0.15	0.02* (-.001 to .06)	0.059	591	0.04**(.005 to .06)	0.03	-0.00 (-.06 to .06)	0.96	593
Subtraction, and less	0.03** (.002 to .06)	0.04	0.02** (.007 to .04)	0.02	592	0.06** (.006 to .10)	0.03	0.01 (-.06 to .08)	0.82	593
Addition	0.04* (-.0008 to .09)	0.053	0.03* (-.006 to .07)	0.09	592	0.105** (.03 to .18)	0.01	0.01 (-.09 to .10)	0.90	593

^a^ Value represents coefficient on treatment variable for grade 5. 95% confidence interval in parentheses.

^b^ Value represents coefficient on treatment variable for grades 3 and 4. 95% confidence interval in parentheses.

***P < 0.01,

**P < 0.05,

*P < 0.10 based on clustered standard errors.

In MP, increases occur in the percent of children who can do addition (11 percent points; 27% of baseline of 41 percent), subtraction and less (6 percent points; 30% of baseline of 20 percent), multiplication and less (4 percent points; 44% of baseline of 9 percent), and division and less (2 percent points; 31% of baseline of 6 percent) in grade 5.

Increases occur in the share of children in grades 3 and 4 able to write (3 percent points, 55% of baseline of 5 percent) and read (5 percent points, 50% of baseline of 10 percent) in UP. It is important to note the size of percent impacts is generally large due to low levels of the absolute learning outcomes at baseline. At midline impacts on learning were fewer and only on reading in grade 3 in UP as well as MP [32].

#### School councils’ participation and awareness

The difference in differences analysis was done using mean response at the GP level. Analysis was not possible at the individual member level in MP as school council elections were held anew during the course of intervention due to change in election rules that reduced the council term to one year. In UP 60% of original council members remained at follow-up, while 40% were new due to routine member turnover.

School councils became more functional after the campaign (Table 4). In UP, there is 12% (0.14 of an average of 1.20 meetings at baseline) increase in the number of school council meetings, 8% (4 percent points of 52 percent at baseline) increase in attendance in meetings, 18% (8 percent points of 44 percent at baseline) increase in member participation in school visits, and 14% (0.25 of an average of 1.77 members attending school visits at baseline) increase in total number of members attending school visits. At midline, the only impacts observed in UP were increases in the number of council meetings (25%) and member participation in school visits (25%) [32]. Although fewer impacts were observed at midline, their higher magnitudes compared to end line could perhaps be explained by two-fifths new council members in end line survey. In MP, an 11% (0.21 of an average of 1.92 visits at baseline) increase occurred in the number of school visits by council members and 14% (9 percent points of 63 percent at baseline) increase in member participation in school visits. At midline, no impact was observed on school council variables in MP [32].

**Table 4:** Difference-in-differences linear regression results in MP and UP where change in school council outcome from baseline to follow-up is dependent variable.

State→	UP			MP		
School council outcome	Treatment-control (95%CI)	P	n	Treatment-control (95%CI)	P	n
Number of meetings	0.14* (.003 to .28)	0.047	553	0.05 (-.08 to .18)	0.41	523
Share attended meeting	0.04*** (.02 to .06)	0.00	553	0.00 (-.04 to .03)	0.95	533
Number of school visits	0.15 (-.21 to .52)	0.35	554	0.21* (-.009 to .44)	0.06	533
Share attended school visits	0.08** (.006 to .14)	0.04	554	0.09** (.03 to .15)	0.01	533
Total number who attended school visits	0.25* (-.04 to .53)	0.08	554	0.41 (-.49 to 1.32)	0.32	518

Value represents coefficient on treatment variable. 95% confidence interval in parentheses.

***P < 0.01,

**P < 0.05,

*P < 0.10 based on clustered standard errors.

In both states, there is an increase in members’ awareness of school accounts and of oversight roles vis-à-vis teachers, although the increase is more in UP compared to MP (S5 Table).

Impact on participation and awareness is larger on councils with higher proportion of high caste members (S6 and S7 Tables, S3 Appendix). Analyzing by member type, the impact tends to be greater on chair and secretary members of councils than on other parent members. This suggests low caste and parent members experienced smaller gains in participation.

### Findings on impact in Karnataka

#### Teacher effort

In Karnataka, the intervention is associated with no change in teacher attendance or activity between baseline and follow-up (Table 5). This is similar to the finding at midline when no impact on teacher effort was observed, and can be because teacher effort was high at baseline both in absolute terms and in comparison to the other two states; almost 90 percent of teachers were present and 80 percent of those present were teaching.

**Table 5:** Difference-in-differences linear regression results in Karnataka where change in outcome from baseline to follow-up is dependent variable.

State→	Karnataka		
**Teacher effort outcome**	Treatment-control (95%CI)	P	n
Attendance all teachers	-0.02 (-.04 to .01)	0.26	483
Activity all teachers	-0.02 (-.06 to .02)	0.29	483
**Learning outcome**	Treatment-control (95%CI)	P	n
Read sentences and words	-0.03 (-.09 to .04)	0.44	401
Write sentences and words	-0.01 (-.07 to .05)	0.77	402
Divide, and less	0.08** (.01 to .15)	0.03	401
Multiply, and less	0.08** (.003 to .16)	0.04	400
Subtraction, and less	0.08** (.001 to .16)	0.047	401
Addition	0.04 (-.04 to .11)	0.28	400
**School council outcome**	Treatment-control (95%CI)	P	n
Number of meetings	-0.05 (-.31 to .21)	0.66	358
Share attended meeting	-0.08* (-.18 to .01)	0.10	359
Number of school visits	0.01 (-.22 to .25)	0.88	355
Share attended school visits	-0.02 (-.11 to .08)	0.71	355
Total number who attended school visits	-0.58 (-1.48 to .31)	0.18	358

Value represents coefficient on treatment variable. 95% confidence interval is in parentheses.

***P < 0.01,

**P < 0.05,

*P < 0.10 based on clustered standard errors.

The impacts for Karnataka are for the two treatments pooled together. When an additional treatment dummy variable is introduced to represent the differential effect of the second treatment, it is not significantly different from zero for the teacher effort and learning variables (S8 Table). This is similar to the findings of no additional effect of the second treatment at midline [32].

#### Learning

We find a consistent impact mainly in the share of children acquiring mathematics competencies (Table 5), similar to results in MP and UP. Increases are observed in the percent of children who can do subtraction and less (8 percentage points; 15% of 52 percent at baseline), multiplication and less (8 percentage points; 24% of 33 percent at baseline), and division and less (8 percentage points; 29% of 28 percent at baseline). There is no change in the share of children able to write or read. In comparison, at midline increases were observed on one competency each in math (multiplication and less) and language (writing) [32].

#### School councils’ participation and awareness

There is no overall change in school council members’ participation associated with the intervention and in fact a decrease in the reported attendance in meetings (Table 5). These findings could reflect new member elections that took place soon after the second round of intervention, not leaving enough time for member participation to change. These findings are similar to those at midline when no change in member participation was observed [32]. But there is an increase in members’ awareness of school accounts and oversight roles vis-à-vis other roles and responsibilities such as preparing schemes to improve school quality and undertaking school visits (S5 Table).

### Qualitative findings on the process of change

#### Focus group discussions

Findings from MP and UP are aggregated in S9 Table. A focus group meeting had 12 participants on average. 63% (509/804) of focus group participants remembered the campaign. Of those who remembered, 72% had discussed the disseminated information with others in the village, and 59% said the discussion went on for weeks. The most frequently-mentioned topics of discussion were learning and teaching: all of the groups mentioned these among the main themes discussed, and 76% listed them ahead of other topics.

At least 33% reported they had taken up issues of teacher presence and learning with those responsible for service delivery in schools (S9 Table, column 2). More than 70% raised the issues with teachers, almost 30% with school council chairs and almost none with other council members. More than 75% went with others rather than alone to talk to this person. When asked about the reaction of the person with whom the issue had been raised, at least a third encountered an angry reaction and also about a third had difficulty discussing these concerns with the person they had raised them with.

More than 60% of focus group participants reported using the learning assessment booklet to assess learning levels and after assessing, about one-fourth raised it as an issue with a service provider. About 82% percent raised it with a teacher, 17% with the school committee chair and none with other council members. More than 75% went with others rather than alone to talk to this person.

When asked why bigger changes did not occur in learning or teacher effort, prominent themes mentioned in the responses were “teacher is dominating,” “it is difficult to talk to the teacher,” “teacher does not listen,” and “teacher does not care.” Among low caste groups, “people are afraid to talk to the teacher” was also mentioned. When asked how the information campaign can be made more effective, the dominant response (73%) was to have more frequent meetings to disseminate information.

#### Interviews of school council members

Council members discussed the information widely, 87% had discussed the information with others and 72% said the discussion went on for weeks (S9 Table, column 3). When asked the three main important issues discussed, 83% cited learning, 64% cited teaching and 63% cited other issues. About 39% raised learning and 27% raised teacher attendance issues with teachers or council chairs. About 78% of members assessed learning levels via the tool. After assessing, 43% discussed it with the teacher (83%) and/or chair (21%). Unlike the focus groups, most members neither faced any anger nor found it difficult to discuss the issue with the person they went to.

Members perceived an improvement in committee meetings, which were now more inclusive and frequent after the campaign. When asked if the campaign made a difference to council meetings, 71% of members reported an improvement. When we asked what improved about meetings, main responses were “meetings are more frequent,” “learning is discussed,” “more members attend,” and “all are informed of when a meeting will take place.” They reported taking specific actions to monitor the schools such as checking school timings and teacher presence, and assessing grade appropriate learning levels. 76% of members reported attempting to assess learning levels in schools, 48% tried to verify teacher presence or school timings by visiting the school, and 25% directly talked to the teacher about his or her teaching.

## Discussion and conclusion

We find that providing information in a structured and repeated manner to communities enhanced the delivery of teacher effort in public school classrooms and improved learning. Improvements occurred in teacher effort, learning and functioning of school committees. The impacts were larger in UP and MP and much smaller in Karnataka possibly because the states had different starting points. Baseline outcomes were much higher in Karnataka indicating greater efficiency in delivery. Impacts were in general larger or broader than those observed in the midline survey that measured impact 2–4 months after the initial intervention [32].

In MP and UP, the impact on teacher effort was primarily for civil-service teachers who have permanent jobs and minimal incentive to work. These effects were 23% increase in teacher attendance in UP, and 16% increase in teacher attendance and 43% increase in teacher activity in MP. Among civil service teachers, the impact was greater for high caste, male and more educated teachers. Low teacher effort for these categories of teachers is a robust finding in the empirical literature for developing countries and attributed to their more powerful social status [10, 37, 40]. This suggests greater impact of the campaign precisely where the accountability problem is more serious. No impact was observed in Karnataka where nearly 90% of teachers were present and 80% of those present were teaching at baseline, leaving little scope for impact. The differential impact can also be because unlike in the other two states, school councils do not have direct control over teacher effort–they neither track attendance nor teacher tenure (S1 Appendix).

On learning outcomes, in MP and UP a consistent result was increase in the shares of children acquiring mathematics competencies. The magnitudes of the percentage increases were much larger, due to low baseline levels of learning, compared to absolute increases in shares which were more modest in magnitude. In Karnataka, the impact occurred in mathematics competencies as in the other two states. This implies that classroom teaching was possibly impacted, although teacher effort as measured in the survey did not change in the state. Not much improvement was observed in language competencies except in UP. The modest impacts on language observed at midline were not sustained in MP and Karnataka. Although the impacts on learning, particularly in math, are larger than those observed at midline, the lack of wider or even larger impacts on learning is attributed to at least two factors. First and perhaps more important, teaching skills and teacher preparation in content and pedagogy are inadequate [1, 41]. Increased teacher presence and teaching will not translate into large learning gains if teaching is ineffective [1]. Second, learning is cumulative and students may need exposure to the intervention for a longer period. Research suggests it can take 5–8 years of implementation of a school-community based intervention, that aims to strengthen accountability, to observe changes in the more difficult to modify indicators such as learning [24].

In terms of process outcomes, in MP and UP school councils were more active after the campaign. Council members reported more meetings and school visits. The greater number of changes in participation and awareness outcomes of school council members in UP relative to MP can be partly due to a differences in the structure, roles and responsibilities of councils (S1 Appendix). VECs in UP are smaller village-level bodies elected for 5 years while PTAs in MP are large school-level committees elected annually. Group size and length of interaction between members are likely to matter for participation. Coordination is difficult in larger teams where the share of influence of each team member is low and can reduce the motivation for individual effort. Also when the council’s term is short, members may not put their weight behind issues that will find resolution beyond their tenure. In both states, gains in council members’ participation varied by caste and position in the council, indicating exclusion for those with less influence. In Karnataka there was no impact on the functioning of school councils in terms of member participation. This might be a result of council elections held too close to the follow-up survey, not leaving enough time for members’ participation to change. Overall, the findings suggest that (a) decentralization to communities is meaningless unless communities at least know what oversight roles they have; (b) providing information to unaware communities can play a useful role in changing behavior (community participation and teacher effort) and learning outcomes, particularly in lagging states; (c) impacts on behavior and learning tend to broader or larger when measured at end line after the campaign sustained over two rounds compared to those measured in a shorter follow up at midline after one round of intervention; and (d) the heterogeneity in impact by state depending on existing level of baseline capacity and nature of devolution emphasizes the role of local context in the results of a RCT. It suggests the usefulness of geographic dispersion of sites and a need for caution before generalizable conclusions are made.

Focus group discussions highlight the mechanisms of change and barriers that remained. First, the information provided was valued given the degree of discussion and monitoring it engendered. Second, learning was important to parents given their involvement in using the learning assessment tool and conferring with service providers. Third, the campaign spurred community action. Many parents actively brought up teaching and learning issues with teachers and councils. Change seems to have occurred through communities discussing and bringing-up issues with service providers. Two critical suggestions made by focus groups are indicative: first, organize a large meeting where the entire community is present and second, provide information regularly. Barriers to collective action are also apparent. The main obstacle to change identified was the powerful status of teachers. Teachers tend to enjoy protection from local elites [40] and therefore it is important that any information provided is not appropriated.

The strengths of this study are that it used a structured, practical and easily replicable intervention, had a rigorous design and broad geographic coverage. Information that becomes common knowledge can help citizens demand services from providers, especially in the context of decentralized control over public services. The study’s results build on previous work [31, 32] and suggest that the design of information provision as well as follow up period may be important.

Key features of the information program can be useful to policy-makers in designing similar programs. Empowering communities with information requires resources, but can still be cost-effective in improving service delivery. The cost of running the campaign was approximately $149 per village cluster yearly. More than 70% of India’s public education budget is teacher salaries [42]. High absenteeism and low effort among teachers result in huge wastage of public resources. We calculated roughly how much the increased attendance from the campaign was worth when valued at teacher costs per day, using a conservative estimate of the salary of a public school teacher (S10 Table). The value of reclaimed public resources through greater teacher presence alone was 22–37 times more than the cost of the campaign, even without taking account of the benefits of improved learning.

Changing behavior to improve learning outcomes in schools requires time. In a meta-analysis of the effectiveness of school-based management models in the US that reviewed 232 studies looking at 29 types of such programs, the number of years of program implementation was a significant predictor of the magnitude of impact on student achievement [43]. This makes sense because such interventions need to challenge unequal societal power structures and institutional inertia to succeed [44, 45]. An important caveat in interpreting our results is that the impact of such an intervention on learning is mediated by crucial factors outside its scope such as teaching effectiveness. While it is intuitive that enhancing teacher effectiveness is key to improve learning, the example of improvement in education outcomes in the state of Delhi in India shows different interventions including building active and participatory school councils and community can be used together as part of systemic reform to transform school education [46, 47].

## Supporting information

S1 Appendix Background on decentralization in basic education in the study states. (DOCX)

S2 Appendix Example of roles and responsibilities of school committees in Madhya Pradesh (MP). (DOCX)

S3 Appendix Definition of low caste groups. (DOCX)

S1 Checklist Inclusivity in global research. (DOCX)

S1 Table Share of rural, literate and poor populations in India, MP, UP and Karnataka. (DOCX)

S2 Table Baseline characteristics by randomized treatment status in MP, UP and Karnataka. Mean values represent shares except indicated otherwise. Sample size is in parentheses when differs due to missing values. Karnataka did not have contract teachers in public schools during the study period.
(DOCX)

S3 Table Baseline school council awareness outcomes by treatment status in MP, UP and Karnataka. Mean values represent shares except indicated otherwise. School council variables represent member response aggregated at village cluster level by member type (chair, secretary, parent member). Sample size is in parentheses in cell when differs due to missing values.
(DOCX)

S4 Table Difference-in-differences linear regression where change in teacher effort outcome from baseline to follow-up is dependent variable, MP and UP. Sample consists of civil-service teachers by characteristics. Value represents coefficient on treatment variable. 95% confidence interval in parentheses.
(DOCX)

S5 Table Difference-in-differences linear regression where change in school council awareness outcome from baseline to follow-up is dependent variable, MP, UP and Karnataka. Value represents coefficient on treatment variable. 95% confidence interval in parentheses.
(DOCX)

S6 Table Difference-in-differences linear regression where change in school council outcome from baseline to follow-up is dependent variable, UP. Value represents coefficient on treatment variable. 95% confidence interval in parentheses.
(DOCX)

S7 Table Difference-in-differences linear regression where change in school council outcome from baseline to follow-up is dependent variable, MP. Value represents coefficient on treatment variable. 95% confidence interval in parentheses.
(DOCX)

S8 Table Difference-in-differences linear regression results where change in outcome from baseline to follow-up is dependent variable, Karnataka. Value represents coefficient on treatment variable. 95% confidence interval in parentheses. Since the two treatments differ in one dimension only and are otherwise identical, the table reports the average impact of the two treatments pooled compared with the control group and additional impact of the second treatment compared with the first.
(DOCX)

S9 Table Focus group discussions, MP and UP. (DOCX)

S10 Table Approximate cost-benefit calculations of (increased teacher effort due to) the intervention in MP and UP. Number of civil-service primary school teachers is taken from National University of Education Planning and Administration (NUEPA), 2009 [S10.1]. Attendance is valued at teacher cost for the total number of civil service teachers in the state. In this case teacher cost is taken as the monthly average salary of Indian Rupees 15,000 for a teacher in each state [39]. Cost of the campaign is calculated for each state using the total number of *gram panchayats* (GPs), 52002 in UP and 22931 in MP [23] and per GP cost of $141 of the campaign. US$ 1 = Indian Rupees (Rs.) 47 as of March 2010.
(DOCX)
