# Supplementary material for: Does information improve service delivery? A randomized trial in education in India
Source: PLoS One. 2023 Mar 15;18(3):e0280803. doi: 10.1371/journal.pone.0280803 (PMC10016677; doi:10.1371/journal.pone.0280803)
Supplement: S1 Appendix — (DOCX) [file pone.0280803.s001.docx]

**S1 Appendix. Background on decentralization in basic education in the study states.**

1. **The Constitutional amendment for devolution**

For many states in India, the 73rd amendment in1992 to the constitution flagged off community interventions in local public service delivery systems. It conferred constitutional status on local governance, with a view to giving communities key role in such governance through transfer of powers, authority and responsibilities in several matters including education and health. The amendment, which instructed states to devolve finances and control over public services to local communities, however left the onus on the structure and degree of control to individual states. Similar to other public services, in the area of basic education most states chose to work through a committee at the school or village level, typically comprising of head teachers, teachers, parents and other community members. This is not unlike the school-based management (SBM) approach followed in other countries.

SBM has been in place in countries with diverse educational systems, such as Australia, Canada, Israel and US- going back 30 years in some cases to relatively recent reforms in several developing countries. SBM can be broadly defined as decentralization of authority from the central or state governments to the school level and such reforms are far from uniform, shaped by the broader national policy and the social context in which they are created. Typically, the devolution of decision-making has two dimensions to it: the extent of autonomy devolved, and the people to whom the authority for decision-making is devolved. Most SBM reforms involve some sort of transfer of responsibility and decision making- usually the responsibility of school operations- to a combination of head teachers, teachers, parents and other community members. Most programs work through a school committee, which may: monitor school performance, for example, in test scores or teacher and student attendance; appoint or dismiss teachers; ensure that teacher salaries are paid on time; approve school budgets; and examine financial statements.

1. **Specifics of local control in basic education in the study states during study period**

Availability of and access to public education services are largely determined by policy at the state level. As mentioned in the main text, states in India differ in the extent and structure of the devolution of control to communities in public services including primary education. Local governance in public schools is organized to function through school councils (also referred to as committees), but the structure and mandate of the councils differ across states. We describe below how the devolution of control is organized to function through school committees in the three study states. There are similarities and differences across states in the structure and mandate of these institutions (school councils) created for local governance of schools.

*Madhya Pradesh (MP)*

In MP, a Parent-Teacher-Association (PTA) is mandatory in every government school, and all parents are obligated to be members. The PTA is expected to operate primarily through the school council which is a 14 member PTA executive committee with a one-year team, although all parents are supposed to participate in decision-making. The PTA elects a parent as the executive committee’s chair from its members, and its secretary is the head teacher. The chair and the secretary jointly operate the school account.

The school account receives annual government grants for repair and maintenance, school development, teacher learning materials (TLMs), school uniforms for students, civil works, and mid-day meals.

The school councils are to monitor learning and teacher performance, and to manage and oversee school funding coming to the school and village government accounts. Council members can visit schools anytime and register any grievance with the district or block education offices.

Public schools in MP have two types of teachers—contract, and civil service teachers. The former are hired on a 3-year renewable contract by the elected government at the block level. In monitoring teacher performance, the school council in MP is mandated to verify teacher attendance in order for the release of teachers’ monthly salaries.

*Uttar Pradesh (UP)*

In UP, in every village cluster (GP) a school council (referred to as village education committee (VEC)) is mandatory. There is one committee for all primary and junior public schools in the GP. The VEC comprises of 5 members, and is chaired by the elected head (*Gram Pradhan*) of the local village government. Other members include the senior-most head teacher who is the secretary and three parents of students. The council has a five-year term, except parent membership can cease if the child is no longer in school. New parent members then replace the old ones. The council chair and the school head teacher jointly operate the school account.

As in MP, the school account receives annual government grants for repair and maintenance, school development, TLMs, school uniforms, and civil works. Additionally in UP, the village government account, co-signed by the school council chair, receives stipends and mid-day meal funds meant for public schools.

The VEC is to monitor learning and teacher performance, and to manage and oversee school funding coming to the school and village government accounts. Council members can visit schools and register any grievance with the district or block education offices.

Public schools in UP have contract and civil service teachers. The VEC is empowered to select contract teachers and review their performance on an annual basis to decide contract renewals. In selecting contract teachers, councils are required to follow state guidelines outlining eligibility criterion. With a two-thirds majority, the council can remove a contract teacher any time during the contract tenure.

*Karnataka*

Every government school is mandated to have a School Development and Monitoring Committee (SDMC). An SDMC has 9 members, including the head teacher of the primary school, parents, and elected representatives of the village government. A member‘s term is up to 3 years. A parent member is elected as the SDMC chair, and the head teacher is the secretary of the committee. The chair and the secretary jointly operate the school account (the type of funds received are similar to those in MP schools).

SDMC is mandated with monitoring learning, and managing and overseeing school funding. The committee is expected to hold a meeting of all parents every 3 months to discuss learning achievements. It is also expected to monitor teacher performance.

Primary schools in Karnataka have only civil service teachers; there are no contract teachers. Unlike the other two states, SDMC does not have any explicit control over teachers; however as in the other two states it can inspect schools and register grievance with district/block education offices.

1. **Summary of differences in structure and mandate of school councils across the study states**

The following is a broad summary of the differences in the structure and mandate of school councils across the three states. 1.) MP and Karnataka have put in place school-level councils with relatively shorter terms (1 year and 3 year, respectively), while UP has a GP-level council, with a longer term of 5 years, that oversees all schools within the administrative territory of the GP. 2.) In MP the council is embedded in the general body of all parents in the school, unlike in the other two states which have no explicitly defined general body other than the body of all voters in the GP (called *Gram Sabha*). 3.) The council size is larger in MP (14 members) and Karnataka (9-14 members) compared to UP (5 members) as also is the number of parent members on the council. 4.) The council is chaired by a parent in MP and Karnataka and by the elected head of the village government in UP. 5.) Relative to Karnataka, MP and UP have devolved greater control to these bodies with respect to teachers. In MP the councils verify teachers‘ attendance in order for teachers to receive their salary, and in UP they make decisions on hiring and renewal of contract teachers.
