# Supplementary material for: Does information improve service delivery? A randomized trial in education in India
Source: PLoS One. 2023 Mar 15;18(3):e0280803. doi: 10.1371/journal.pone.0280803 (PMC10016677; doi:10.1371/journal.pone.0280803)
Supplement: S2 Appendix — (DOCX) [file pone.0280803.s002.docx]

**S2 Appendix. Example of roles and responsibilities of school committees in Madhya Pradesh (MP).**

We describe below a sample of the information provided to the community regarding the main roles and responsibilities of the parent-teacher association (PTA):

• Ensure that the school is functioning well

• Prepare schemes for improving schools

• Ensure that all children in ages 5-14 are enrolled in school

• Ensure that children attend school regularly

• Look after any construction in the school, repairs and management of existing schools

• Manage and monitor the funds coming into the school education account

• Decide how money is to be spent based on the school‘s needs and give consent for use of

funds

• Monitor the distribution of textbooks, scholarships and uniforms

• Implement the mid-day meal program. Monitor the quality of food served

• Ensure that children are learning at appropriate levels for their grade

• Ensure that teachers come to school regularly and teach properly

• PTA chair verifies every teacher‘s attendance monthly by signing on teacher‘s attendance

sheet. Can stop any teachers‘ salary by not signing on teachers‘ attendance sheet if teacher

does not come regularly

• Complain to the Block/District Education Office or Cluster Resource Center and recommend disciplinary action if dissatisfied with teacher (examples: if teachers do not come regularly or do not carry out their duties appropriately)

• Ensure at least 200 teaching days per school year, and at least 5 hours of teaching on an

average per day
