# Supplementary material for: Does information improve service delivery? A randomized trial in education in India
Source: PLoS One. 2023 Mar 15;18(3):e0280803. doi: 10.1371/journal.pone.0280803 (PMC10016677; doi:10.1371/journal.pone.0280803)
Supplement: S3 Appendix — (DOCX) [file pone.0280803.s003.docx]

**S3 Appendix. Definition of low caste groups.**

We refer to Scheduled caste (SC) and Scheduled tribe (ST) populations as socially disadvantaged or low caste groups in the paper. They are at the bottom of the caste and socio-economic hierarchy in India. The scheduled castes (SCs) and scheduled tribes (STs) are two groupings of historically disadvantaged populations given recognition in the Indian constitution. They are recognized as disadvantaged due to extreme social, economic and educational backwardness arising from the traditional practice of untouchability (SCs) and geographic isolation and little social contact with the community at large (STs).^[[1]](#footnote-1)^ Non-low caste population groups are referred to as high caste in the paper. Disadvantaged or low caste groups depend disproportionately on public services, compared with higher castes who are more often able to afford private services such as physicians and private schools.

1. For definitions of SC and ST groups, see <https://dmeo.gov.in/sites/default/files/2022-10/Thematic-report_Social-Inclusion_14102022-%20Final.pdf>, <https://ncst.nic.in/content/frequently-asked-questions> [↑](#footnote-ref-1)
