# Supplementary material for: Does information improve service delivery? A randomized trial in education in India
Source: PLoS One. 2023 Mar 15;18(3):e0280803. doi: 10.1371/journal.pone.0280803 (PMC10016677; doi:10.1371/journal.pone.0280803)
Supplement: S1 Table — (DOCX) [file pone.0280803.s005.docx]

**S1 Table. Share of rural, literate and poor populations in India, MP, UP and Karnataka.**

|  | % Rural^a^ | % Literate^a^ | % Literate, rural^a^ | % Poor^b^ | % Poor^c^ | % Poor rural^c^ |
| --- | --- | --- | --- | --- | --- | --- |
| India | 69 | 74 | 68 | 55 | 28 | 37^c^ |
| MP | 72 | 71 | 64 | 69.5 | 41 | 46^d^ |
| UP | 78 | 70 | 65 | 70 | 41 | 44^d^ |
| Karnataka | 61 | 76 | 69 | 46 | 17 | 19^d^ |

^a^ Census of India 2011 [23].

^b^ % Poor is based on multi-dimensional poverty index reported in Oxford Poverty and Human Development Initiative (OPHI) 2010 [34].

^c^ % Poor is based on multi-dimensional poverty index reported in Oxford Poverty and Human Development Initiative (OPHI) 2021 [35].

^d^ % Poor is based on multi-dimensional poverty index reported in NITI Aayog 2021 [S1.1].

# References

S1.1 NITI Aayog. India National Multidimensional Poverty Index: Baseline Report. Government of India. 2021. Available from: [https://www.niti.gov.in/sites/default/files/2021- 11/National_MPI_India-11242021.pdf](https://www.niti.gov.in/sites/default/files/2021-11/National_MPI_India-11242021.pdf)
