# Supplementary material for: Does information improve service delivery? A randomized trial in education in India
Source: PLoS One. 2023 Mar 15;18(3):e0280803. doi: 10.1371/journal.pone.0280803 (PMC10016677; doi:10.1371/journal.pone.0280803)
Supplement: S2 Table — Mean values represent shares except indicated otherwise. Sample size is in parentheses when differs due to missing values. Karnataka did not have contract teachers in public schools during the study period. (DOCX) [file pone.0280803.s006.docx]

**S2 Table. Baseline characteristics by randomized treatment status in MP, UP and Karnataka.**

| State→ | UP | |  | MP | |  | Karnataka | | |
| --- | --- | --- | --- | --- | --- | --- | --- | --- | --- |
|  | Control  Mean | Treatment  Mean | P^a^ | Control  Mean | Treatment  Mean | P^a^ | Control  Mean | Treatment  Mean | P^a^ |
| **Student characteristics** | n =4404 | n =4427 |  | n =4096 | n =4152 |  | n =1580 | n =3302 |  |
| Age (years) | 8.76  (n=4371) | 8.69  (n=4420) | 0.37 | 9.01 (n=4097) | 8.89^*^  (n=4154) | 0.08 | 10.07 | 10.09 | 0.77 |
| Male | 0.49 | 0.49 | 0.51 | 0.52 (n=4098) | 0.50  (n=4154) | 0.59 | 0.50 | 0.53^*^ | 0.06 |
| Mother literate | 0.21 (n=4269) | 0.20  (n=4296) | 0.49 | 0.14 | 0.12 | 0.32 | 0.37 | 0.36 | 0.90 |
| Father literate | 0.61  (4124) | 0.58^*^  (n=4192) | 0.05 | 0.46 (n=4097) | 0.45  (n=4153) | 0.74 | 0.57 | 0.55 | 0.63 |
| Land owned by family (acres) | 1.17  (n=4384) | 1.11  (n=4416) | 0.43 | 2.49  (n=3624) | 2.53  (n=3706) | 0.86 | 3.40  (n=1453) | 3.46  (n=3020) | 0.85 |
| **School characteristics** | n =100 | n =100 |  | n =99 | n =100 |  | n =70 | n =140 |  |
| Enrollment (number) | 185.88 | 170.09  (n=99) | 0.12 | 124.24  (n=98) | 113.20 | 0.27 | 128 | 141 | 0.42 |
| Student-teacher ratio | 69.87 | 61.68 (n=99) | 0.15 | 54.98 (n=97) | 56.73 | 0.70 | 27 | 26  (n=139) | 0.70 |
| Schools with toilet | 0.39 | 0.28 | 0.15 | 0.38 | 0.37 | 0.83 | 67 | 74 | 0.55 |
| Schools with drinking water | 0.82 | 0.84 | 0.68 | 0.70 | 0.74 | 0.54 | 67 | 65  (n=136) | 0.73 |
| Schools with playground | 0.88 | 0.84 | 0.46 | 0.48 | 0.58 | 0.13 | 38 (n=68) | 49  (n=138) | 0.24 |
| Schools with electricity | 0.00 | 0.01 | 0.35 | 0.09 | 0.04 | 0.28 | 55 (n=69) | 62  (n=138) | 0.31 |
| Blackboards (number) | 4.00 | 3.51 | 0.11 | 3.45 | 3.19 | 0.28 | 5.4 (n=68) | 6  (n=138) | 0.38 |
| **Teacher characteristics** | n =335 | n =311 |  | n =233 | n =229 |  | n =281 | n =586 |  |
| Age (years) | 38.09 | 38.42 | 0.66 | 38.36 | 38.84 | 0.67 | 38.70 | 39.15 | 0.50 |
| Male | 0.60 (n=336) | 0.61  (n=312) | 0.72 | 0.81 | 0.80 | 0.77 | 61 | 58 | 0.50 |
| Civil-service teacher | 0.57 (n=334) | 0.61 | 0.20 | 0.86 | 0.84 | 0.60 | 100 | 100 | - |
| Contract teacher | 0.43 (n=334) | 0.39 | 0.20 | 0.14 | 0.16 | 0.60 | - | - | - |
| Completed high school | 0.41 | 0.43 | 0.67 | 0.47 | 0.48 | 0.84 | 73 | 71 | 0.82 |
| Completed college | 0.31 | 0.34 | 0.27 | 0.27 | 0.33 | 0.39 | 24 | 26 | 0.79 |
| Completed higher than college | 0.28 | 0.23^*^ | 0.08 | 0.26 | 0.19 | 0.33 | 2.85 | 2.73 | 0.92 |
| Pre-service training | 0.58 | 0.59 | 0.70 | 0.37 | 0.35 | 0.77 | 91 | 94 | 0.13 |
| Teaching experience (years) | 10.86 (n=334) | 10.95 | 0.90 | 13.33 (n=232) | 14.19  (n=228) | 0.40 | 11.95 | 12.23 | 0.68 |
| Commute to school (kilometers) | 5.88 | 6.68 | 0.40 | 8.99  (n=218) | 9.30  (n=225) | 0.84 | 9.59 | 10.04 | 0.73 |
| **School council size** | n=98 | n=98 |  | n=99 | n=100 |  | n=65 | n=138 |  |
| Number of council members | 5.78 | 5.23 | 0.101 | 11.02 | 11.22 | 0.38 | 8.95 | 8.86 | 0.64 |

Mean values represent shares except indicated otherwise. Sample size is in parentheses when differs due to missing values. Karnataka did not have contract teachers in public schools during the study period. ^a^ P value for difference between treatment and control groups based on clustered standard errors. ***P < 0.01, **P < 0.05, *P < 0.10
