# Supplementary material for: Does information improve service delivery? A randomized trial in education in India
Source: PLoS One. 2023 Mar 15;18(3):e0280803. doi: 10.1371/journal.pone.0280803 (PMC10016677; doi:10.1371/journal.pone.0280803)
Supplement: S3 Table — Mean values represent shares except indicated otherwise. School council variables represent member response aggregated at village cluster level by member type (chair, secretary, parent member). Sample size is in parentheses in cell when differs due to missing values. (DOCX) [file pone.0280803.s007.docx]

**S3 Table. Baseline school council awareness outcomes by treatment status in MP, UP and Karnataka.**

| State→ | UP | | | MP | | | Karnataka | | |
| --- | --- | --- | --- | --- | --- | --- | --- | --- | --- |
| **School council members’ awareness** | Control  mean | Treatment  mean |  | Control  mean | Treatment  mean |  | Control  mean | Treatment  mean |  |
| **Awareness of accounts** | n =288 | n=273 | P^a^ | n =271 | n =270 | P^a^ | n =152 | n=348 | P^a^ |
| Knowledge of school accounts | .59 | .55 | 0.15 | .61 | .59 | 0.69 | 0.75 | 0.72 | 0.56 |
| Knowledge of stipend account | .54 | 50 | 0.23 | .36 | .30^*^ | 0.06 | 0.75 | 0.75 | 0.92 |
| Knowledge of mid-day meal account | .48 | .42^*^ | 0.09 | .39  (n=260) | .36  (n=254) | 0.41 | 0.71 | 0.69 | 0.66 |
| **Awareness of roles** | n=290 | n=289 | P^a^ | n=271 | n=270 | P^a^ |  |  |  |
| Knowledge of teacher aspects of roles and responsibilities (mentioned at least one role-monitoring attendance, ensuring teacher teaches, complain if teacher does not perform, any other) | .33 | .38 | 0.19 | .33 | .32 | 0.86 | 0.56 | 0.61 | 0.35 |
| Knowledge of other aspects of roles and responsibilities (mentioned at least one role- inspecting schools, managing civil works, preparing schemes for school improvement, managing school funds) | .42 | .41 | 0.72 | .45 | .45 | 0.96 | 0.65 | 0.66 | 0.88 |
| Knowledge of entitlements aspects of roles and responsibilities (mentioned at least one-ensuring textbooks, scholarships, quality mid-day meals, uniforms reach students) | .28 | .33 | 0.13 | .51 | .48 | 0.26 | 0.65 | 0.71 | 0.18 |

Mean values represent shares except indicated otherwise. Sample size is in parentheses in cell when differs due to missing values.

^a^ P value for difference between treatment and control groups based on clustered standard errors.

***P < 0.01, **P < 0.05, *P < 0.10
