# Supplementary material for: Does information improve service delivery? A randomized trial in education in India
Source: PLoS One. 2023 Mar 15;18(3):e0280803. doi: 10.1371/journal.pone.0280803 (PMC10016677; doi:10.1371/journal.pone.0280803)
Supplement: S4 Table — Sample consists of civil-service teachers by characteristics. Value represents coefficient on treatment variable. 95% confidence interval in parentheses. (DOCX) [file pone.0280803.s008.docx]

**S4 Table. Difference-in-differences linear regression where change in teacher effort outcome from baseline to follow-up is dependent variable, MP and UP.**

| State→ | UP | | | | MP | | | |
| --- | --- | --- | --- | --- | --- | --- | --- | --- |
| Dependent variable → (Follow-up - baseline) | Attendance | | Activity | | Attendance | | Activity | |
| Sample of civil-service teachers↓ | Treatment-Control (95% CI) | n | Treatment-Control (95% CI) | n | Treatment-Control (95% CI) | n | Treatment-Control (95% CI) | n |
| High caste | 0.15^**^ (.03 to .27) | 170 | -0.01 (-.02 to .01) | 170 | 0.09^**^ (.01 to .16) | 172 | 0.14^**^ (.02 to .25) | 172 |
| Low caste | -0.01 (-.63 to .60) | 28 | 0.02 (-.29 to .33) | 28 | 0.19^**^ (.006 to .36) | 76 | 0.09 (-.12 to .31) | 76 |
| Male | 0.13^*^ (-.02 to .28) | 134 | 0.01 (-.04 to .07) | 134 | 0.13^***^ (.08 to .18) | 202 | 0.14^***^ (.06 to .22) | 202 |
| Female | 0.09^***^ (.07 to .10) | 64 | -0.03 (-.14 to .07) | 64 | 0.09 (-.10 to .28) | 49 | 0.11 (-.08 to .31) | 49 |
| Experience ≥ median | 0.04 (-.09 to .18) | 99 | 0.01 (-.06 to .07) | 99 | 0.12^**^ (.04 to .19) | 163 | 0.14^***^ (.07 to .19) | 163 |
| Experience < median | 0.23^**^ (.06 to .40) | 99 | -0.02 (-.11 to .05) | 99 | 0.09 (-.03 to .21) | 87 | 0.09 (-.02 to .20) | 87 |
| Education >college | 0.27^***^ (.13 to .40) | 56 | -0.02 (-.09 to .03) | 56 | 0.06 (-.29 to .41) | 50 | 0.25^*^ (-.03 to .53) | 50 |
| Education ≤college | 0.03 (-.16 to .22) | 142 | -0.01 (-.03 to .01) | 142 | 0.12^**^ (.007 to .22) | 201 | 0.09^**^ (.007 to .18) | 201 |

Sample consists of civil-service teachers by characteristics. Value represents coefficient on treatment variable. 95% confidence interval in parentheses.

***P < 0.01, **P < 0.05, *P < 0.10 based on clustered standard errors.
