# Supplementary material for: Does information improve service delivery? A randomized trial in education in India
Source: PLoS One. 2023 Mar 15;18(3):e0280803. doi: 10.1371/journal.pone.0280803 (PMC10016677; doi:10.1371/journal.pone.0280803)
Supplement: S5 Table — Value represents coefficient on treatment variable. 95% confidence interval in parentheses. (DOCX) [file pone.0280803.s009.docx]

**S5 Table. Difference-in-differences linear regression where change in school council awareness outcome from baseline to follow-up is dependent variable, MP, UP and Karnataka.**

|  | UP | | | MP | | | Karnataka | | |
| --- | --- | --- | --- | --- | --- | --- | --- | --- | --- |
| School council awareness | Treatment-Control (95% CI) | P | n | Treatment-Control (95% CI) | P | n | Treatment-Control (95% CI) | P | n |
| Knowledge of school accounts | 0.12^***^ (.08 to .15) | 0.00 | 570 | -0.01 (-.07 to .04) | 0.59 | 533 | 0.13^***^(.06 to .19) | 0.00 | 360 |
| Knowledge of stipend account | 0.09^**^ (.01 to .16) | 0.03 | 570 | 0.07 (-.03 to .18) | 0.15 | 533 | 0.04 (-.05 to .14) | 0.33 | 360 |
| Knowledge of mid-day meal account | 0.08^***^ (.03 to .11) | 0.00 | 570 | 0.07^**^ (.005 to .12) | 0.04 | 506 | 0.00 (-.11 to .11) | 0.97 | 360 |
| Knowledge of roles and responsibilities vis-à-vis teachers | 0.11^***^ (.04 to .16) | 0.00 | 571 | 0.02^*^ (-.002 to .03) | 0.08 | 533 | 0.04 (-.06 to .15) | 0.41 | 360 |
| Knowledge of other roles and responsibilities | 0.03^***^ (.02 to .04) | 0.00 | 571 | 0.06 (-.10 to .21) | 0.42 | 533 | 0.16^*^ (.03 to .28) | 0.02 | 359 |
| Knowledge of roles and responsibilities vis-à-vis entitlements | -0.05 (-.11 to .01) | 0.12 | 571 | 0.05 (-.02 to .12) | 0.16 | 533 | -0.03 (-.16 to .10) | 0.62 | 360 |

Value represents coefficient on treatment variable. 95% confidence interval in parentheses.

***P < 0.01, **P < 0.05, *P < 0.10 based on clustered standard errors.
