# Supplementary material for: Does information improve service delivery? A randomized trial in education in India
Source: PLoS One. 2023 Mar 15;18(3):e0280803. doi: 10.1371/journal.pone.0280803 (PMC10016677; doi:10.1371/journal.pone.0280803)
Supplement: S6 Table — Value represents coefficient on treatment variable. 95% confidence interval in parentheses. (DOCX) [file pone.0280803.s010.docx]

**S6 Table.** **Difference-in-differences linear regression where change in school council outcome from baseline to follow-up is dependent variable, UP.**

|  | UP | | | | | | | |
| --- | --- | --- | --- | --- | --- | --- | --- | --- |
| School council  sample → | Proportion of high caste members in school council | | | | Member type | | | |
|  | Above median | | Below median | | Chair/  Secretary | | Parent | |
| Dependent variable  (Follow-up - baseline)↓ | Treatment-control | n | Treatment-control | n | Treatment-control | n | Treatment-control | n |
| Number of Meetings | 0.25^**^  (.04 to .45) | 304 | 0.07 (-.16 to .31) | 249 | 0.26^*^(-.006 to .53) | 362 | 0.12 (-.05 to .30) | 375 |
| Attended meeting | 0.11^***^ (.05 to .16) | 304 | -0.02 (-.06 to .02) | 249 | 0.07^**^ (.01 to .12) | 362 | 0.04 (-.02 .10) | 375 |
| Number of Inspections | 0.28 (-.29 to .84) | 305 | 0.02 (-.22 to .25) | 249 | 0.20 (-.20 to .59) | 363 | 0.24 (-.29 to .77) | 375 |
| Attended inspection | 0.10 (-.07 to .28) | 305 | 0.05 (-.03 to.12) | 249 | 0.09^*^  ( -.0006 to .18) | 363 | 0.12^**^ (.01 to .22) | 375 |
| Persons present at inspection | 0.61^**^ (.06 to 1.16) | 305 | -0.15 (-.56 to .24) | 249 | 0.30 (-.13 to .73) | 363 | 0.38^**^ (.04 to .70) | 375 |
| Knowledge of school accounts | 0.14^***^  (.06 to .21) | 307 | 0.10^*^  (-.02 to .24) | 263 | 0.15^***^ (.13 to .17) | 373 | 0.12^***^  (.05 to .18) | 386 |
| Knowledge of stipend account | 0.08 (-.05 to .21) | 307 | 0.11^**^ (.01 to .20) | 263 | 0.10^*^ (-.02 to .21) | 373 | 0.10^**^ (.02 to .16) | 386 |
| Knowledge of mid-day meal account | 0.10^***^ (.05 to .13) | 307 | 0.07^*^ (-.01 to .16) | 263 | 0.11^***^(.04 to .16) | 373 | 0.07^**^ (.02 to .11) | 386 |

Value represents coefficient on treatment variable. 95% confidence interval in parentheses.

***P < 0.01, **P < 0.05, *P < 0.10 based on clustered standard errors.
