# Supplementary material for: Does information improve service delivery? A randomized trial in education in India
Source: PLoS One. 2023 Mar 15;18(3):e0280803. doi: 10.1371/journal.pone.0280803 (PMC10016677; doi:10.1371/journal.pone.0280803)
Supplement: S7 Table — Value represents coefficient on treatment variable. 95% confidence interval in parentheses. (DOCX) [file pone.0280803.s011.docx]

|  | MP | | | | | | | |
| --- | --- | --- | --- | --- | --- | --- | --- | --- |
| School council  Sample → | Proportion of high caste members in school council | | | | Member type | | | |
|  | Above median | | Below median | | Chair/  Secretary | | Parent | |
| Dependent variable  (Follow-up - baseline)↓ | Treatment-control | n | Treatment-control | n | Treatment-control | n | Treatment-control | n |
| Number of Meetings | 0.03 (-.16 to .23) | 249 | 0.07 (-.03 to .18) | 274 | 0.14 (-.04 to .32) | 326 | 0.08 (-.08 to .24) | 349 |
| Attended meeting | 0.07^***^ (.04 to .09) | 252 | -0.06  (-.12 to .02) | 281 | 0.00 (-.03 to .02) | 335 | 0.03 (-.04 to .09) | 357 |
| Number of Inspections | 0.42^***^ (.23 to .59) | 252 | 0.06 (-.32 to .44) | 281 | 0.36^***^ ( .14 to .58) | 335 | 0.25^*^(-.01 to .51) | 357 |
| Attended inspection | 0.13^**^ (.05 to .19) | 252 | 0.06 (-.05 to .17) | 281 | 0.15^***^ (.06 to .24) | 335 | 0.08^*^ (-.01 to .17) | 357 |
| Persons present at inspection | 0.38 (-1.00 to 1.76) | 244 | 0.46 (-.44 to 1.37) | 274 | 0.86 (-.55 to 2.27) | 320 | 0.04 (-.69 .78) | 354 |
| Knowledge of school accounts | 0.07 (-.04 to .18) | 252 | -0.08^**^ (-.14 to -.01) | 281 | -0.01 (-.08 to .07) | 335 | -0.03 (-.10 to .04) | 357 |
| Knowledge of stipend account | 0.04 (-.03 to .10) | 252 | 0.09 (-.09 to .27) | 281 | 0.11^*^ (-.02 to .24) | 335 | 0.10^**^ (.01 to .18) | 357 |
| Knowledge of mid-day meal account | 0.17^**^ (.02 to .32) | 238 | -0.03 (-.15 to .09) | 268 | 0.08 (-.03 to .20) | 309 | 0.10^***^ (.05 to .13) | 341 |

**S7 Table.** **Difference-in-differences linear regression where change in school council outcome from baseline to follow-up is dependent variable, MP.**

Value represents coefficient on treatment variable. 95% confidence interval in parentheses.

***P < 0.01, **P < 0.05, *P < 0.10 based on clustered standard errors.
