# Supplementary material for: Does information improve service delivery? A randomized trial in education in India
Source: PLoS One. 2023 Mar 15;18(3):e0280803. doi: 10.1371/journal.pone.0280803 (PMC10016677; doi:10.1371/journal.pone.0280803)
Supplement: S8 Table — Value represents coefficient on treatment variable. 95% confidence interval in parentheses. Since the two treatments differ in one dimension only and are otherwise identical, the table reports the average impact of the two treatments pooled compared with the control group and additional impact of the second treatment compared with the first. (DOCX) [file pone.0280803.s012.docx]

**S8 Table.** **Difference-in-differences linear regression results where change in outcome from baseline to follow-up is dependent variable, Karnataka.**

| **State→** | **Karnataka** | | | | |
| --- | --- | --- | --- | --- | --- |
| **Teacher effort outcome** | Treatment-control (95%CI) | P | Additional effect of Treatment 2 (95%CI) | P | n |
| Attendance all teachers | -0.01 (-.05 to .03) | 0.63 | -0.01 (-0.05 to 0.03) | 0.48 | 483 |
| Activity all teachers | -0.02 (-.10 to .06) | 0.54 | 0.00 (-0.11 to 0.11) | 0.97 | 483 |
| **Learning outcome** | Treatment-control (95%CI) | P | Additional effect of Treatment 2 (95%CI) | P | n |
| Read sentences and words | -0.05 (-.15 to .04) | 0.26 | 0.05 (-0.06 to 0.16) | 0.32 | 401 |
| Write sentences and words | -0.02 (-.11 to .06) | 0.57 | 0.03 (-0.08 to 0.14) | 0.60 | 402 |
| Divide, and less | 0.08^**^ (.02 to .15) | 0.02 | -.006 (-.08 to 0.07) | 0.84 | 401 |
| Multiply, and less | 0.09^**^ (.02 to .16) | 0.02 | -0.01 (-.09 to 0.06) | 0.70 | 400 |
| Subtraction, and less | 0.10^**^ (.007 to .19) | 0.04 | -0.03 (-0.09 to 0.03) | 0.27 | 401 |
| Addition | 0.05 (-.04 to .14) | 0.21 | -0.02 (-0.09 to 0.04) | 0.38 | 400 |
| **School Council outcome** | Treatment-control (95%CI) | P | Additional effect of Treatment 2 (95%CI) | P | n |
| Number of meetings | -0.19 (-.51 to .12) | 0.20 | 0.29 (-0.09 to 0.68) | 0.12 | 358 |
| Share attended meeting | -0.15^**^ (-.28 to -.03) | 0.02 | 0.15^*^ (-0.03 to 0.33) | 0.09 | 359 |
| Number of school visits | -0.02 (-.30 to .25) | 0.84 | 0.08 (-0.22 to 0.39) | 0.55 | 355 |
| Share attended school visits | -0.04 (-.13 to .06) | 0.42 | 0.04 (-0.07 to 0.15) | 0.44 | 355 |
| Total number who attended school visits | -0.84 (-1.96 to .27) | 0.12 | 0.54 (-1.11 to 2.20) | 0.48 | 358 |

Value represents coefficient on treatment variable. 95% confidence interval is in parentheses. Since the two treatments differ in one dimension only and are otherwise identical, the table reports the average impact of the two treatments pooled compared with control group and additional impact of the second treatment compared with the first.

***P < 0.01, **P < 0.05, *P < 0.10 based on clustered standard errors.
