# Supplementary material for: Does information improve service delivery? A randomized trial in education in India
Source: PLoS One. 2023 Mar 15;18(3):e0280803. doi: 10.1371/journal.pone.0280803 (PMC10016677; doi:10.1371/journal.pone.0280803)
Supplement: S9 Table — (DOCX) [file pone.0280803.s013.docx]

**S9 Table. Focus group discussions, MP and UP.**

| **Questions** | **Focus group participants’ response (%)** | **School council members’ response (%)** |
| --- | --- | --- |
| ***Discussion of information***  Did you discuss the information with others in the village | Yes (72 %) | Yes (87%) |
| How long did the discussions continue | Weeks (59%) Months (8%) Days (33%) | Weeks (72%) Months (9%) Days (19%) |
| What were the three main issues discussed | Learning (83%) Teaching (64%) Other (63%) | Learning (83%) Teaching (64%) Other (63%) |
| ***Issues raised with those responsible for service delivery***  After the campaign, did you ever raise teacher attendance as an issue with the teacher/chair/school committee/an education official | Yes (33 %) | Yes (27 %) |
| If yes, who did you raise it with | Teacher (70%), chair (29%), other school committee member (0%), education official (1%) | Teacher (76%), chair (24%), other school committee member (0%), education official (0%) |
| If yes, did you go alone or with others to talk to this person | With others (88%), alone (12%) | With others (29%), alone (71%) |
| Did the person you raised the issue with get angry and was it difficult to talk to this person | Person got angry (48%), difficult to talk to this person (32%) | Person got angry (5%), difficult to talk to this person (0%) |
| After the campaign, did you ever raise learning as an issue with the teacher/chair/school committee/an education official | Yes (39%) | Yes (39%) |
| If yes, who did you raise it with | Teacher (82%), chair (17%), other school committee member (1%), education official (0%) | Teacher (97%), chair (3%), other school committee member (0%), education official (0%) |
| If yes, did you go alone or with others to talk to this person | With others (77%), alone (24%) | With others (7%), alone (93%) |
| Did the person you raised the issue with get angry and was it difficult to talk to this person | Person got angry (33%), difficult to talk to this person (32%) | Person got angry (3%), difficult to talk to this person (3%) |
| After the campaign, did you ever use the learning assessment tool to assess learning of your child | Yes (62%) | Yes (78%) |
| After assessing, did you ever discuss it with the teacher/chair/school committee/an education official | Yes (24%) | Yes (43%) |
| If yes, who did you discuss with | Teacher (82%), chair (17%), other school committee member (0%), education official (0%) | Teacher (83%), chair (21%), other school committee member (0%), education official (0%) |
| If yes, did you go alone or with others to talk to this person | With others (77%), alone (22%) | With others (8%), alone (92%) |
| Did the person you raised the issue with get angry and was it difficult to talk to this person | Person got angry (56%), difficult to talk to this person (23%) | Person got angry (8%), difficult to talk to this person (4%) |
| Did the campaign make any difference to school committee meetings | - | Yes (71%) |
| ***Specific questions on participation***  Did you ever assess learning in school | - | Yes (76%) |
| Did you ever talk to the teacher about his or her teaching | - | Yes (25%) |
| Did you verify teacher attendance and school timings by making a visit to the school | - | Yes (48%) |
