# Supplementary material for: Does information improve service delivery? A randomized trial in education in India
Source: PLoS One. 2023 Mar 15;18(3):e0280803. doi: 10.1371/journal.pone.0280803 (PMC10016677; doi:10.1371/journal.pone.0280803)
Supplement: S10 Table — Number of civil-service primary school teachers is taken from National University of Education Planning and Administration (NUEPA), 2009 [S10.1]. Attendance is valued at teacher cost for the total number of civil service teachers in the state. In this case teacher cost is taken as the monthly average salary of Indian Rupees 15,000 for a teacher in each state [39]. Cost of the campaign is calculated for each state using the total number of gram panchayats (GPs), 52002 in UP and 22931 in MP [23] and per GP cost of $141 of the campaign. US$ 1 = Indian Rupees (Rs.) 47 as of March 2010. (DOCX) [file pone.0280803.s014.docx]

**S10 Table. Approximate cost-benefit calculations of (increased teacher effort due to) the intervention in MP and UP.**

| State | Number of days in a school year | Total number of civil service teachers at primary level 2007-08^a^ | Mean baseline attendance of civil service teachers | Increase in attendance due to the intervention | Value of increased attendance per year in Indian rupees Rs. (US $ equivalent^d^)^b^ | Cost of the intervention per year in Indian rupees Rs. (US $ equivalent^d^)^c^ |
| --- | --- | --- | --- | --- | --- | --- |
| MP | 220 | 3,04,268 | 64% | 17% (11 percentage points) | Rs. 6.02 billion  (US$ 128 million) | Rs. 0.16 billion  (US$ 3.4 million) |
| UP | 200 | 3,28,212 | 61% | 23% (14 percentage points) | Rs. 8.27 billion (US$ 176 million) | Rs. 0.36 billion  (US$ 7.7 million) |

^a^Number of civil-service primary school teachers is taken from National University of Education Planning and Administration (NUEPA), 2009 [S10.1].

^b^Attendance is valued at teacher cost for the total number of civil service teachers in the state. In this case teacher cost is taken as the monthly average salary of Indian Rupees 15,000 for a teacher in each state [39].

^c^Cost of the campaign is calculated for each state using the total number of *gram panchayats* (GPs) 52,002 in UP and 22,931 in MP [23] and per GP cost of $141 of the campaign.

^d^ US$ 1= Indian Rupees (Rs.) 47 as of March 2010.

# References

S10.1 NUEPA (National University of Education Planning and Administration). Elementary Education in India. Where do we stand? Ministry of Human Resource Development, Government of India, New Delhi. 2009.
